# Supplementary material for: Humoral immune response to SARS-CoV-2 and endemic coronaviruses in urban and indigenous children in Colombia
Source: Commun Med (Lond). 2023 Oct 20;3:151. doi: 10.1038/s43856-023-00376-9 (PMC10589283; doi:10.1038/s43856-023-00376-9)
Supplement: Supplementary file 1 — Supplementary Information [file 43856_2023_376_MOESM1_ESM.pdf]

Supplementary Information - Humoral immune response to SARS-CoV-2 and endemic coronaviruses in urban and indigenous children in Colombia

Nathalie Verónica Fernández Villalobos[1,*], Patrick Marsall[2,*] , Johanna Carolina Torres Páez[1], Julia Strömpl[3], Jens Gruber[2], Martín Lotto Batista[1,4], Daria Pohl[3], Gustavo Concha[5], Hagen Frickmann[6,7], Fernando Pio de la Hoz Restrepo[8], Nicole Schneiderhan-Marra[2], Gérard Krause[3,9,10,11], Alex Dulovic[2], Monika Strengert[3,11,&,**], Simone Kann[12,&]

[1]Department of Epidemiology, PhD Programme, Helmholtz Centre for Infection Research (HZI), Braunschweig-Hannover, Germany.

[2]Multiplex Immunoassays, NMI Natural and Medical Sciences Institute at the University of Tübingen (NMI), Reutlingen, Germany.

[3]Department of Epidemiology, Helmholtz Centre for Infection Research (HZI), Braunschweig, Germany.

[4]Global Health Resilience, Barcelona Supercomputing Center (BSC), Barcelona, Spain.

[5]Organization Wiwa Yugumaiun Bunkauanarrua Tayrona (OWYBT), Department Health Advocacy, Valledupar, Colombia.

[6]Department of Microbiology and Hospital Hygiene, Bundeswehr Hospital Hamburg, Hamburg, Germany.

[7]Institute for Medical Microbiology, Virology and Hygiene, University Medicine Rostock, Rostock, Germany.

[8]Universidad Nacional de Colombia, Facultad de Medicina, Departamento de Salud Pública, Bogotá, Colombia.

[9]Hannover Medical School, Hannover, Germany.

[10]German Centre for Infection Research (DZIF), partner site: Braunschweig-Hannover, Germany.

[11]TWINCORE, Centre for Experimental and Clinical Infection Research, a joint venture of the Hannover Medical School and the Helmholtz Centre for Infection Research, Hannover, Germany.

[12]Medical Mission Institute, Würzburg, Germany.

** Corresponding author
E-Mail: Monika.Strengert@helmholtz-hzi.de (MS)

[*] These authors contributed equally
[&] These authors jointly supervised this work

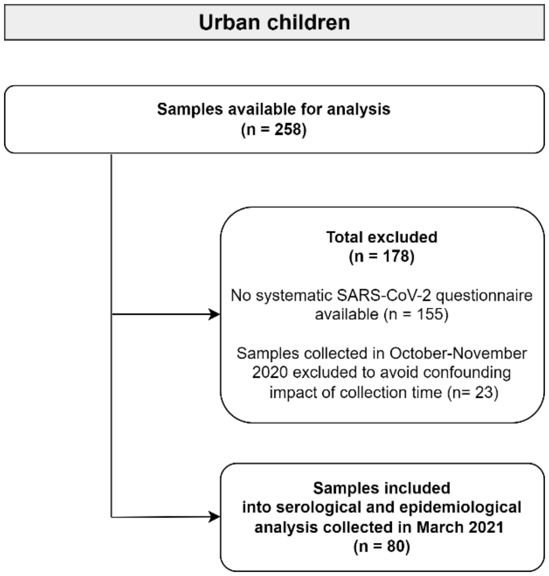

Supplementary Figure 1. Sample selection strategy for analysis of SARS-CoV-2 and endemic coronavirus antibody responses in urban children

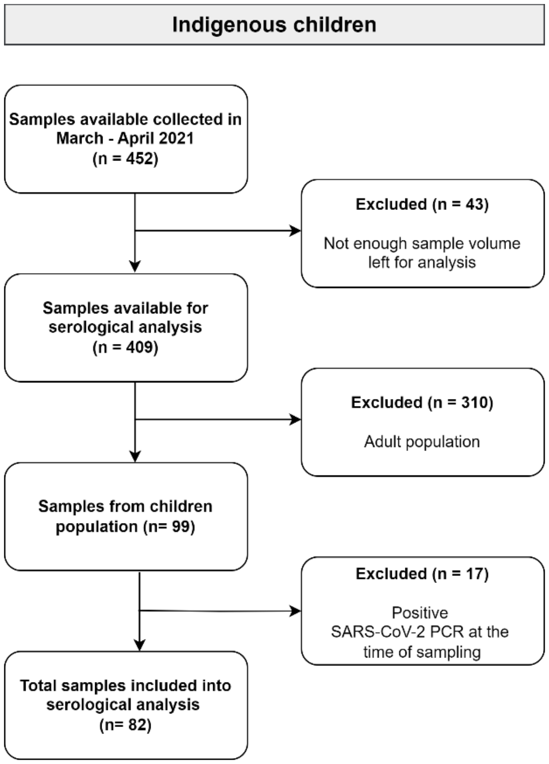

Supplementary Figure 2. Sample selection strategy for analysis of SARS-CoV-2 and endemic coronavirus antibody responses in indigenous children

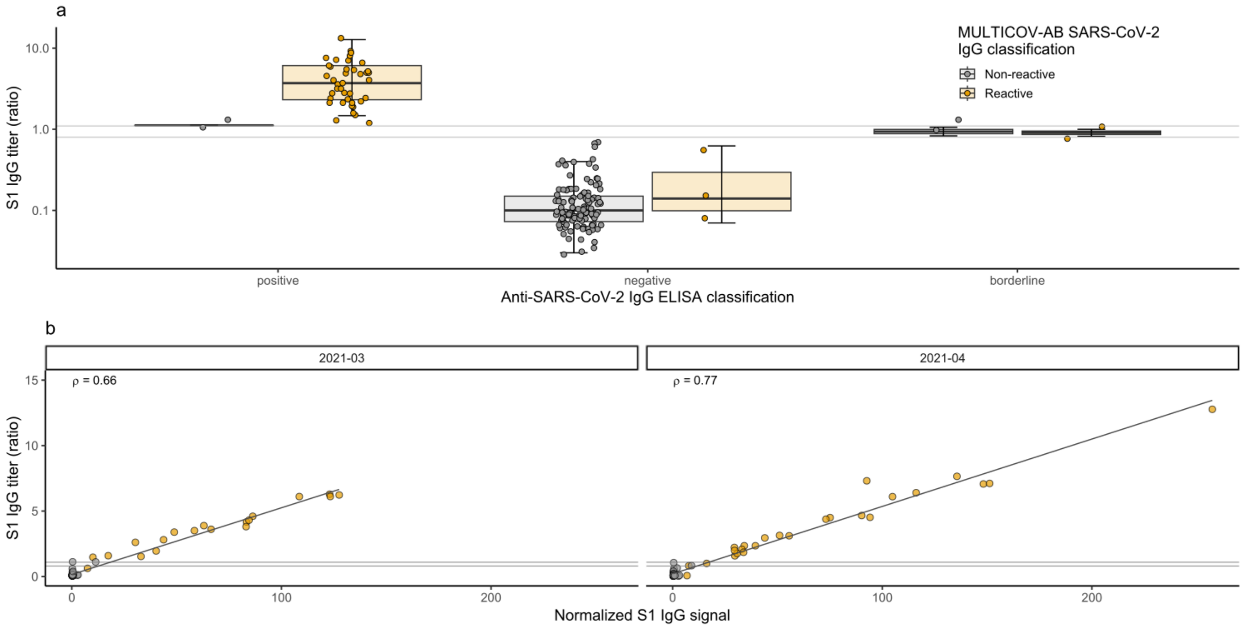

Supplementary Figure 3. Comparison between SARS-CoV-2 IgG ELISA and MULTICOV-AB results in the study population (n=162)

a. Spike S1-specific serum IgG levels plotted as semi-quantitative S/CO ratio on the y-axis were categorized into the respective qualitative ELISA results (non-reactive, borderline, reactive). Boxes represent the median, 25th and 75th percentiles, whiskers show the largest and smallest non-outlier values. Outliers were determined by 1.5 times IQR. **b.** SARS-CoV-2 S1 IgG titers measured by MULTICOV-AB (x-axis) and EUROIMMUN ELISA (y-axis) were plotted for correlation analysis by Spearman coefficient ρ. Samples in graph b were additionally split based on collection time. Borderline results (ratio: ≥0.8 - <1.1) in the ELISA are located within the grey lines (a, b). Qualitative MULTICOV-AB results per individual sample are indicated by yellow- (reactive) or grey (non-reactive) -colored symbols (a, b).

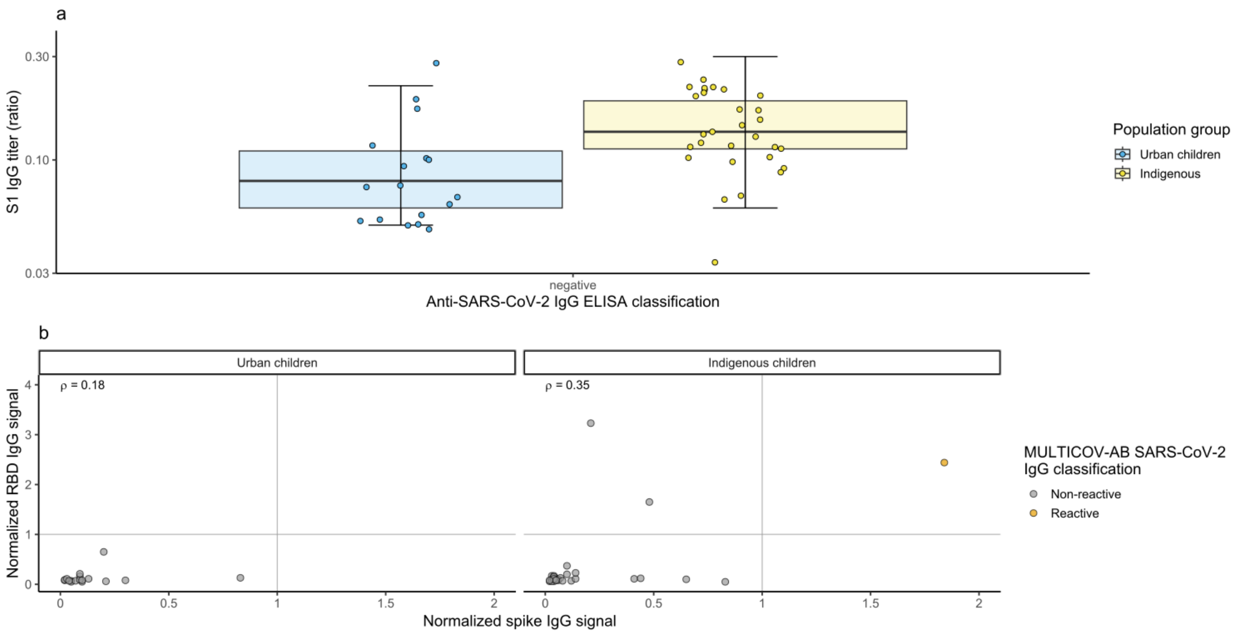

Supplementary Figure 4. Measurement of pre-pandemic indigenous and urban sera samples

Samples from urban children (n=17) and from the Wiwa community (n=30) collected in 2018 and 2019 were measured as specificity control with the EUROIMMUN ELISA (a) and MULTICOV-AB (b). S/CO ratios for the indicated antigens were plotted to illustrate value distribution. Reactivity in the EUROIMMUN ELISA is defined as S/CO ratio ≥1.1 (a) or for MULTICOV-AB as dual S/CO ratio ≥1.0 (b, grey lines) for the indicated antigens. For correlation analysis between antigens, Spearman coefficient ρ was used (b). Qualitative MULTICOV-AB results per individual sample are indicated by yellow- (reactive) or grey (non-reactive) -colored symbols (b). Boxes represent the median, 25th and 75th percentiles. Whiskers show the largest and smallest non-outlier values (a).

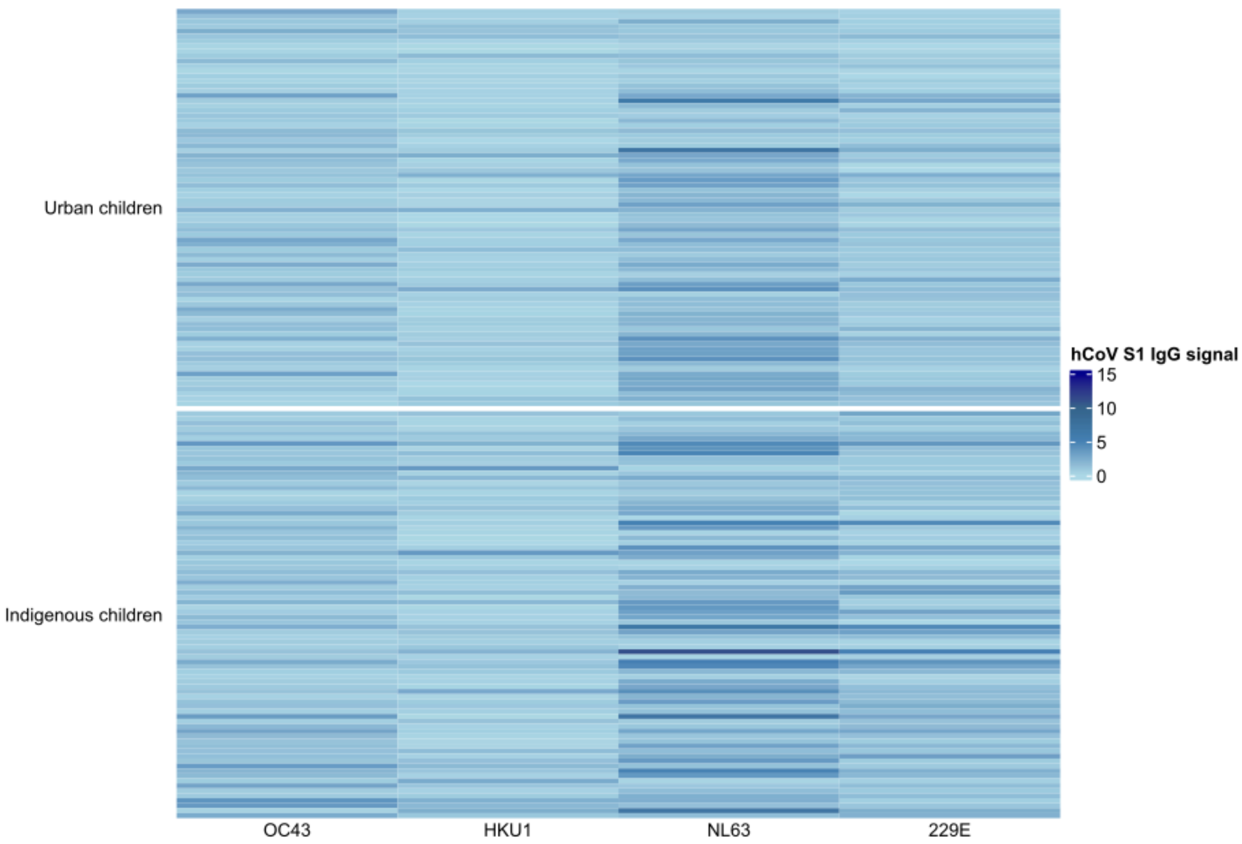

Supplementary Figure 5. Humoral immune response towards the endemic human coronaviruses in urban and indigenous children

S1 IgG responses towards the endemic coronaviruses OC43, HKU1, NL63 and 229E were measured in children from Bogotá (n=80) and from the indigenous Wiwa (n=82) using MULTICOV-AB. Normalized MFI ratios are plotted as heatmap with color shades from light to dark blue to display increasing S1 IgG signal ratios.

Supplementary Table 1. Exclusion factors from the study for children and their companions living in Bogotá

**Comorbidities or medical conditions**
Companions above the age of 60
Hypertension
Obesity
Malnutrition
Pregnancy
Predisposition for bleeding or blood clots
Any form of immune disorder
Metabolic syndromes (thyroid disease, severe liver or kidney disease, diabetes)
Neurologic disorders, cognitive deficits
Untreated asthma or other pulmonary diseases

Supplementary Table 2. Characteristics of urban children from Bogotá

Characteristics of the participants		80 (100%) All participants: n (%)	63 (78.8%) SARS-CoV-2 IgG non-reactive: n (%)*	17 (21.2%) SARS-CoV-2 IgG reactive: n (%)*	p-value (Fisher's Exact Test)
Socio-economic conditions					
Age in years: Median (IQR)		11 (9-14)	11 (9-14)	11 (8-15)	---§
Sex	Male	37 (46.3)	28 (44.4)	9 (52.9)	p=0.59
	Female	43 (53.7)	35 (55.6)	8 (47.1)	
BMI (kg/m^2): Median (IQR)		19.35 (16.96-21.36)	18.82 (16.59-21.31)	19.65 (17.98-22.16)	---§
School type	Public	53 (66.3)	40 (63.5)	13 (76.5)	p=0.40
	Private	27 (33.7)	23 (36.5)	4 (23.5)	
Affiliation to the health system	Subsidized	8 (10.0)	8 (12.7)	0 (0.0)	p=0.19
	Contributory	72 (90.0)	55 (87.3)	17 (100.0)	
Socioeconomically strata**	One	4 (5.0)	2 (3.2)	2 (11.8)	p=0.46
	Two	46 (57.5)	36 (57.1)	10 (58.8)	
	Three	29 (36.2)	24 (38.1)	5 (29.4)	
	Four	1 (1.3)	1 (1.6)	0 (0.0)	
Income†	1-2 minimum wages	53 (66.2)	39 (61.9)	14 (82.3)	**p=0.02‡**
	2-6 minimum wages	18 (22.5)	18 (28.6)	0 (0.0)	
	> 6 minimum wages	3 (3.8)	2 (3.2)	1 (5.9)	
	Reply denied	5 (6.2)	4 (6.3)	1 (5.9)	
	Unknown	1 (1.3)	0 (0.0)	1 (5.9)	
Locality	Ciudad Bolívar	29 (36.2)	20 (31.7)	9 (52.9)	p=0.51
	Kennedy	30 (37.5)	25 (39.7)	5 (29.4)	
	Bosa	12 (15.0)	10 (15.9)	2 (11.8)	
	Tunjuelito	9 (11.3)	8 (12.7)	1 (5.9)	
Country	Colombia	79 (98.7)	62 (98.4)	17 (100.0)	p=1.00
	Venezuela	1 (1.3)	1 (1.6)	0 (0.0)	
Variables associated with SARS-CoV-2 infection					
Participants with PCR-confirmed SARS-CoV-2 infection	Yes	6 (7.5)	0 (0.0)	6 (35.3)	**p=<0.001**
	No	74 (92.5)	63 (100)	11 (64.7)	
Contact with probable or confirmed COVID-19 case	Yes	15 (18.7)	8 (12.7)	7 (41.2)	**p=0.01‡**
	No	64 (80.0)	54 (85.7)	10 (58.8)	
	Unknown	1 (1.3)	1 (1.6)	0 (0.0)	
Contact with individuals suffering from COVID-19 related symptoms	Yes	2 (2.5)	1 (1.6)	1 (5.9)	p=0.38
	No	78 (97.5)	62 (98.4)	16 (94.1)	
Members of the family with confirmed SARS-CoV-2 infection	Yes	17 (21.3)	9 (14.3)	8 (47.1)	**p=0.006**
	No	63 (78.7)	54 (85.7)	9 (52.9)	
Healthcare worker in the family	Yes	5 (6.2)	4 (6.3)	1 (5.9)	p=1.00
	No	75 (93.8)	59 (93.7)	16 (94.1)	
Travel history of minor or household member to location with confirmed COVID-19 cases within 14 days before participation	Yes	8 (10.0)	7 (11.1)	1 (5.9)	p=1.00
	No	72 (90.0)	56 (88.9)	16 (94.1)	
Paracetamol use	Yes	13 (16.3)	7 (11.1)	6 (35.3)	**p=0.02**
	No	67 (83.7)	56 (88.9)	11 (64.7)	

* Based on MULTICOV-AB SARS-CoV-2 IgG serostatus
** One corresponds to the lowest and six to the highest strata
† Colombian minimum wage approximate 280 USD
‡ Unknown category and Reply denied category were not included in the calculation
§ Quantitative variables were examined using a logistic regression: Age OR 1.01 per year (95% CI: 0.86-1.19), BMI OR 1.10 per kg/m^2 (95% CI: 0.94-1.31)

Supplementary Table 3. Self-reported symptoms of urban children from Bogotá

**Symptoms**		**80 (100%) All participants n (%)**	**63 (78.8%) SARS-CoV-2 IgG non-reactive n (%) ***	**17 (21.2%) SARS-CoV-2 IgG reactive n (%) ***	**p-value (Fisher's Exact Test)**
Respiratory symptoms					
Fever	No	76 (95.0)	59 (93.6)	17 (100.0)	p=0.57
	Yes	4 (5.0)	4 (6.4)	0 (0.0)	
Nasal congestion	No	64 (80.0)	52 (82.5)	12 (70.6)	p=0.31
	Yes	16 (20.0)	11 (17.5)	5 (29.4)	
Cough	No	75 (93.7)	60 (95.2)	15 (88.2)	p=0.28
	Yes	5 (6.3)	3 (4.8)	2 (11.8)	
Throat pain	No	75 (93.7)	60 (95.2)	15 (88.2)	p=0.28
	Yes	5 (6.3)	3 (4.8)	2 (11.8)	
Breathing difficulties	No	80 (100.0)	63 (100.0)	17 (100.0)	---
Loss of smell or taste	No	79 (98.7)	63 (100.0)	16 (94.1)	p=0.21
	Yes	1 (1.3)	0 (0.0)	1 (5.9)	
Muscle or bone pain	No	78 (97.5)	62 (98.4)	16 (94.1)	p=0.38
	Yes	2 (2.5)	1 (1.6)	1 (5.9)	
Gastrointestinal symptoms					
Nausea	No	75 (93.7)	61 (96.8)	14 (82.3)	p=0.06
	Yes	5 (6.3)	2 (3.2)	3 (17.7)	
Abdominal pain	No	74 (92.5)	60 (95.2)	14 (82.3)	p=0.11
	Yes	6 (7.5)	3 (4.8)	3 (17.7)	
Diarrhea	No	73 (91.2)	58 (92.1)	15 (88.2)	p=0.64
	Yes	7 (8.8)	5 (7.9)	2 (11.8)	

* Based on MULTICOV-AB SARS-CoV-2 IgG serostatus

Supplementary Table 4. MULTICOV-AB antigen panel

Virus	Antigen	Manufacturer	Cat #
SARS-CoV-2	spike trimer	NMI	-
SARS-CoV-2	RBD	NMI	-
SARS-CoV-2	S1 domain	NMI	-
SARS-CoV-2	S2 domain	Sino Biological	40590-V08B
SARS-CoV-2	Nucleocapsid	Aalto Bioreagents	CK 6404-b
HCoV-OC43	S1 domain	NMI	-
HCoV-HKU1	S1 domain	NMI	-
HCoV-NL63	S1 domain	NMI	-
HCoV-229E	S1 domain	NMI	-

Supplementary Table 5. RBDCoV-ACE2 antigen panel

Virus	Antigen	Manufacturer	Cat #
SARS-CoV-2	RBD Wuhan (B.1)	NMI	-
SARS-CoV-2	RBD Delta (B.1.617.2)	NMI	-
SARS-CoV-2	RBD Omicron (BA.1)	Sino Biological	40592-V08H121
SARS-CoV-2	RBD Mu (B.1.621)	NMI	-
SARS-CoV-2	RBD Gamma (P1)	NMI	-

Supplementary Table 6. SARS-CoV-2 seroprevalence adjustment[1] with assay sensitivity and specificity

Assay	Sensitivity	Specificity
MULTICOV-AB	88.3% calculated from 181/205 reconvalescent individuals with a previous PCR-confirmed SARS-CoV-2 infection [2]	100% calculated with samples from 72/72 uninfected individuals [2]
SARS-CoV-2 ELISA (EI 2606-9601G)	80% calculated from 164/205 reconvalescent SARS-CoV-2-infected [2]	97.2% calculated from 70/72 uninfected individuals [2]

Supplementary Table 7. R-packages used for statistical analysis and data visualization

Analysis	R add-on package
Fisher's exact test (function fisher.test())	stats [3]
Generalized Linear Models (GLM) with binomial family, logit link, and Maximum-likelihood (ML) estimation	tidyverse [4], MASS [5]
Seroprevalence adjustment according to Lang and Reiczigel [1]	asht [6]
Fleiss's k statistic with 95% CI	irrCAC [7]
Graphical display	gridExtra [8], ComplexHeatmap [9], ggplot2 [10], cowplot [11], ggpubr [12], ggpmisc [13]

Supplementary Table 8. ACE2 binding inhibition responder rates* towards indicated SARS-CoV-2 RBDs in seropositive** children

% ACE2 binding inhibition responder rates in SARS-CoV-2 seropositive children					
		**RBD B.1**	**RBD ɣ**	**RBD μ**	**RBD o BA.1**
**Urban children (17)**	**Responder (n)**	4	0	0	0
	**Responder (%)**	**23.5**	**0.0**	**0.0**	**0.0**
**Indige-nous children (28)**	**Responder (n)**	7	1	1	0
	**Responder (%)**	**25.0**	**3.6**	**3.6**	**0.0**

* Responders are defined with an ACE2 binding inhibition of >20%

** Based on MULTICOV-AB SARS-CoV-2 IgG serostatus

Supplementary References

1. Lang, Z. & Reiczigel, J. Confidence limits for prevalence of disease adjusted for estimated sensitivity and specificity. *Preventive Veterinary Medicine* **113**, 13-22 (2014).
2. Becker, M.*, et al.* Exploring beyond clinical routine SARS-CoV-2 serology using MultiCoV-Ab to evaluate endemic coronavirus cross-reactivity. *Nature Communications* **12**, 1152-1152 (2021).
3. Team, R.C. R: A Language and Environment for Statistical Computing. (R Foundation for Statistical Computing, Vienna, Austria, 2021).
4. Wickham, H.*, et al.* Welcome to the Tidyverse. *Journal of Open Source Software* **4**, 1686-1686 (2019).
5. Ripley, W.N.V. & B, D. *Modern Applied Statistics with S*, (Springer, New York, 2002).
6. Fay, M.P. asht: Applied Statistical Hypothesis Tests. (2022).
7. Gwet, K.L. & PhD. Computing Chance-Corrected Agreement Coefficients (CAC). (2019).
8. Auguie, B. gridExtra: Miscellaneous Functions for "Grid" Graphics. (2017).
9. Gu, Z., Eils, R. & Schlesner, M. Complex heatmaps reveal patterns and correlations in multidimensional genomic data. *Bioinformatics* **32**, 2847-2849 (2016).
10. Wickham, H. *ggplot2: Elegant Graphics for Data Analysis*, (Springer-Verlag New York, New York, 2016).
11. Wilke, C.O. cowplot: Streamlined Plot Theme and Plot Annotations for 'ggplot2'. (2020).
12. Kassambara, A. ggpubr: 'ggplot2' Based Publication Ready Plots. (2020).
13. Aphalo, P.J. ggpmisc: Miscellaneous Extensions to 'ggplot2'. (2022).
