# Supplementary material for: Humoral immune response to SARS-CoV-2 and endemic coronaviruses in urban and indigenous children in Colombia
Source: Commun Med (Lond). 2023 Oct 20;3:151. doi: 10.1038/s43856-023-00376-9 (PMC10589283; doi:10.1038/s43856-023-00376-9)
Supplement: Supplementary file 3 — Reporting Summary [file 43856_2023_376_MOESM3_ESM.pdf]

nature portfolio

Corresponding author(s): Strengert, COMMSMED-23-0124

Last updated by author(s): 30/06/23

Reporting Summary

Nature Portfolio wishes to improve the reproducibility of the work that we publish. This form provides structure for consistency and transparency in reporting. For further information on Nature Portfolio policies, see our Editorial Policies and the Editorial Policy Checklist.

Statistics

For all statistical analyses, confirm that the following items are present in the figure legend, table legend, main text, or Methods section.

n/a	Confirmed	
☐	☒	The exact sample size (*n*) for each experimental group/condition, given as a discrete number and unit of measurement
☐	☒	A statement on whether measurements were taken from distinct samples or whether the same sample was measured repeatedly
☐	☒	The statistical test(s) used AND whether they are one- or two-sided<br>*Only common tests should be described solely by name; describe more complex techniques in the Methods section.*
☐	☒	A description of all covariates tested
☒	☐	A description of any assumptions or corrections, such as tests of normality and adjustment for multiple comparisons
☐	☒	A full description of the statistical parameters including central tendency (e.g. means) or other basic estimates (e.g. regression coefficient) AND variation (e.g. standard deviation) or associated estimates of uncertainty (e.g. confidence intervals)
☐	☒	For null hypothesis testing, the test statistic (e.g. *F*, *t*, *r*) with confidence intervals, effect sizes, degrees of freedom and *P* value noted<br>*Give P values as exact values whenever suitable.*
☒	☐	For Bayesian analysis, information on the choice of priors and Markov chain Monte Carlo settings
☒	☐	For hierarchical and complex designs, identification of the appropriate level for tests and full reporting of outcomes
☐	☒	Estimates of effect sizes (e.g. Cohen's *d*, Pearson's *r*), indicating how they were calculated

Our web collection on statistics for biologists contains articles on many of the points above.

Software and code

Policy information about availability of computer code

Data collection	Urban children: Self-reported data was collected through a structured questionnaire using REDCap 7.3.6 electronic data capture tools hosted at Unidad de Informática y Comunicaciones - Facultad de Medicina - Universidad Nacional de Colombia.<br>Serological analysis: MULTICOV-AB and RBDCoV-ACE2: xPonent 4.3, SARS-CoV-2 ELISA EUROIMMUN: Tecan iControl
Data analysis	Statistical analysis was performed in RStudio version 4.0.252 using specific R add-on packages as stated in the supplementary material section. Figures were then further edited in Inkscape, if needed. The type of statistical analysis used is listed in the relevant figure legend.<br>SARS-CoV-2 serological assay data was initally analyzed in Excel 2016.

For manuscripts utilizing custom algorithms or software that are central to the research but not yet described in published literature, software must be made available to editors and reviewers. We strongly encourage code deposition in a community repository (e.g. GitHub). See the Nature Portfolio guidelines for submitting code & software for further information.

Data

Policy information about availability of data

All manuscripts must include a data availability statement. This statement should provide the following information, where applicable:

- Accession codes, unique identifiers, or web links for publicly available datasets
- A description of any restrictions on data availability
- For clinical datasets or third party data, please ensure that the statement adheres to our policy

The datasets and data analysis performed in this study will be made available in a public repository upon publication.

Human research participants

Policy information about studies involving human research participants and Sex and Gender in Research.

Reporting on sex and gender	Sex as biological attribute was included in the study. Sex was collected as an observational characteristic with reported confirmation by the legal guardian of the participant.
Population characteristics	Indigenous children: Clinical and epidemiological information on exact areas of origin, age, sex, ethnicity, blood pressure, heart rate, body temperature and blood oxygenation, as well as individually recorded clinical information with a potential relation to a previous or present COVID-19 disease were collected as described in Concha et al. (https://doi.org/10.3390/vaccines9101120).<br>Urban children: Data on demographic (age, sex, country of birth), social (school type, social security affiliation, socio-economical strata, income), clinical (symptoms, previous SARS-CoV-2 PCR results), and exposure variables (healthcare worker in the family, prior travel history to regions with confirmed COVID-19 cases, use of anti-inflammatory medication) were collected for the study population.
Recruitment	Urban children: The present study is an extension to a cross-sectional survey to determine hepatitis A virus and hepatitis E virus seroprevalence in children aged 5-18 years. Recruitment strategy is described in more detail here: https://doi.org/10.3389/fpubh.2023.981172. Briefly, based on the division of Bogotá in 20 localities and 6 stratum areas, a two-stage cluster random sampling was designed considering localities and schools. Localities in Bogotá with all strata (Suba, Usaquén, and Chapinero) and those with low socioeconomic conditions (San Cristóbal, Ciudad Bolívar, Usme, Bosa, and Santa Fe) were considered for taking part in the study. From those pre-identified localities, Ciudad Bolívar and Usaquén and then different schools within those areas were randomly selected. However, as a consequence of the COVID-19 pandemic and the ensuing low numbers of participants, we invited other localities and schools to take part in the study.<br>Indigenous children: Recruitment for this population is described in more detail here: https://doi.org/10.3390/vaccines9101120. Briefly, a study team began to test for SARS-CoV-2 in various Wiwa villages in the north-eastern part of Colombia. The number of inhabitants of these villages was used as a denominator to estimate the proportion of the population addressed. The testing was announced early enough to provide a sufficient time frame for the decision to participate in the study or not.
Ethics oversight	Both studies were performed in line with the Declaration of Helsinki. The study conducted in Bogotá was approved by the Comité De Ética De Investigación De La Facultad De Medicina, Universidad Nacional de Colombia, Bogotá, Colombia (N °.009-125-19 and N°. 011-083) and by the Ethics Committee of Hannover Medical School, Hannover, Germany (Nr.9254_BO_K_2020). The study conducted in the north-eastern Colombian territory was approved by the Ethics Committee for Science of the University Area Andina, Bogotá, Colombia (number 1304211). All participants or their legal representatives provided written informed consent prior to study start. Participation in both studies was voluntary.

Note that full information on the approval of the study protocol must also be provided in the manuscript.

Field-specific reporting

Please select the one below that is the best fit for your research. If you are not sure, read the appropriate sections before making your selection.

☒ Life sciences ☐ Behavioural & social sciences ☐ Ecological, evolutionary & environmental sciences

For a reference copy of the document with all sections, see nature.com/documents/nr-reporting-summary-flat.pdf

Life sciences study design

All studies must disclose on these points even when the disclosure is negative.

Sample size	No formal sample size calculation was carried out for this study.
Data exclusions	Urban children: Children with any predisposition for bleeding, blood clots, cognitive deficits that prevent giving informed assent or consent, or suffering from primary or secondary immunodeficiency were excluded from the study. As a consequence of the COVID-19 pandemic, children and companions exhibiting acute respiratory symptoms or suffering from comorbidities associated with an increased risk of severe COVID-19

	were excluded from the study to prevent SARS-CoV-2 transmissions in the study center. We excluded samples collected not in March and April 2021 as this would introduce an analysis bias based on different collection times.<br>Indigenous children: We excluded 17 children with a SARS-CoV-2 positive PCR / antigen test result at the time of serum sampling from our analysis.
Replication	Samples were measured once.
Randomization	Please refer to recruitment for details about sampling strategy.
Blinding	We conducted an observational study, so blinding was not relevant for the study.

Reporting for specific materials, systems and methods

We require information from authors about some types of materials, experimental systems and methods used in many studies. Here, indicate whether each material, system or method listed is relevant to your study. If you are not sure if a list item applies to your research, read the appropriate section before selecting a response.

Materials & experimental systems

n/a	Involved in the study	
☐	☒	Antibodies
☒	☐	Eukaryotic cell lines
☒	☐	Palaeontology and archaeology
☒	☐	Animals and other organisms
☒	☐	Clinical data
☒	☐	Dual use research of concern

Methods

n/a	Involved in the study	
☒	☐	ChIP-seq
☒	☐	Flow cytometry
☒	☐	MRI-based neuroimaging

Antibodies

Antibodies used	For the SARS-CoV-2 IgG ELISA (cat no: EI 2606-9601G, EUROIMMUN), the supplied, undisclosed antibodies were used according to the user manual.<br>Antibodies used as part of MULTICOV-AB and RBDCoV-ACE2 are listed in the Material and Method section.
Validation	SARS-CoV-2 IgG ELISA (cat no: EI 2606-9601G, EUROIMMUN) was performed according to the supplied manual, which also contains details about how the assay was developped and validated. In addition, each SARS-CoV-2 ELISA plate had to pass assay-specific quality control criteria defined by the manufacturer to be considered a valid run.<br>MULTICOV-AB: Validation of the assay was carried out in: Becker, M., Strengert, M., Junker, D. et al. Exploring beyond clinical routine SARS-CoV-2 serology using MultiCoV-Ab to evaluate endemic coronavirus cross-reactivity. Nat Commun 12, 1152 (2021). https://doi.org/10.1038/s41467-021-20973-3. In addition, to be considered a valid run, conditions as listed in Renk, Dulovic et al. Robust and durable serological response following pediatric SARS-CoV-2 infection. Nat Commun. 2022 Jan 10;13(1):128. doi: 10.1038/s41467-021-27595-9. were used.<br>RBDCoV-ACE2: Validation of this assay was carried out in: Junker, D., Dulovic, A., Becker, M. et al. COVID-19 patient serum less potently inhibits ACE2-RBD binding for various SARS-CoV-2 RBD mutants. Sci Rep 12, 7168 (2022). https://doi.org/10.1038/s41598-022-10987-2
